# Emergence of extensively and pan-drug resistance in clinical bacterial isolates: A systematic scoping review from Ethiopian public health perspective

**DOI:** 10.1371/journal.pntd.0013363

**Published:** 2025-08-28

**Authors:** Muluneh Assefa, Mitkie Tigabie, Azanaw Amare, Getu Girmay, Alene Geteneh, Getnet Ayalew, Sirak Biset, Wesam Taher Almagharbeh

**Affiliations:** 1 Department of Medical Microbiology, School of Biomedical and Laboratory Sciences, College of Medicine and Health Sciences, University of Gondar, Gondar, Ethiopia; 2 Department of Immunology and Molecular Biology, School of Biomedical and Laboratory Sciences, College of Medicine and Health Sciences, University of Gondar, Gondar, Ethiopia; 3 Department of Medical Laboratory Science, College of Health Sciences, Woldia University, Woldia, Ethiopia; 4 Department of Medical and Surgical Nursing, Faculty of Nursing, University of Tabuk, Tabuk, Saudi Arabia; National University of Singapore, SINGAPORE

## Abstract

**Introduction:**

The growing challenge of antimicrobial resistance in Ethiopia and itsprogression towards XDR and PDR has become a critical public health concern. Therefore, thisreview determined the current state of emerging XDR and PDR bacteria, including pre-XDR and XDR-TB, their contributing factors, advancements, and future perspectives against drug-resistant bacteria, as well as their implications for public health and insights for future research.

**Methodology:**

This review followed the Preferred Reporting Items for Systematic Reviews and Meta-Analyses Extension for Scoping Reviews (PRISMA-ScR) guidelines. A systematic search of all available literature was conducted using PubMed/Medline, Scopus, EMBASE, Google Scholar, Hinari, Web of Science, ScienceDirect, Cochrane Library, and African Journals Online databases.This study included original articles published in English that reported XDR and PDR bacteria, Pre-XDR-TB, and XDR-TBb without limit on the study period and publication year. Descriptive statistics were used to summarize the findings.

**Results:**

Twenty-five studies published between 2010 and 2025 were included in this review. Among 5620 bacterial isolates identified,1289 were XDR (22.9%), with the prevalence ranging from 5.7% to 43.2%. A total of 440 bacterial isolates were PDR (9.1%), with its prevalence in individual studies ranged from 0.8% to 19.1%. The most common XDR bacteria identified were *Klebsiella* species; 26.7% (2.8%-84.6%), followed by *E. coli*; 26.4%(14.6%-35.7%), *Acinetobacter* species*;* 24.9%(10.1%-58.3%), and *P. aeruginosa;* 18.7% (2.8%-44.4%). The most frequently identified PDR bacteria were *Acinetobacter* species*;* 17.3% (7.9%-50.0%), followed by *Klebsiella* species*;* 13.7%(2.7%-25.8%), *E. coli*; 10.2%(2.4%-22.6%), and *P. aeruginosa;* 5.7%(4.3%-33.3%). Additionally, from 1419 MDR-TB and 160 TB confirmed cases, Pre-XDR-TB was 3.4% (2.4%-5.7%) and XDR-TB was 1.5%(0.6%-10.0%). These isolates were identified from different clinical specimens, which represents a significant concern in community and hospital settings.

**Conclusion:**

The emergence of XDR and PDR represents a major threat to Ethiopian public health, resulting in increased morbidity, mortality, prolonged hospitalizations, high healthcare costs, and challenged treatment options. Urgent national surveillance and genomic detection of resistance mechanisms are needed to better track the spread of drug-resistant bacteria, promote antimicrobial stewardship, and enhance drug and vaccine trials.

## Author summary

This systematic scoping review addresses the critical public health concern of antimicrobial resistance in Ethiopia, specifically focusing on the emergence of XDR and PDR bacteria, including Pre-XDR and XDR-TB. The review, adhering to PRISMA-ScR guidelines, synthesized findings from 25 studies published between 2010 and 2025. Results indicate that among 5620 bacterial isolates, 22.9% were XDR and 9.1% were PDR. *Klebsiella* species, *E. coli*, *Acinetobacter* species, and *P. aeruginosa* were the most common XDR and PDR bacteria identified. Additionally, pre-XDR-TB was found in 3.4% and XDR-TB in 1.5% of TB confirmed cases. Inappropriate antibiotic use, inadequate sanitation, and agricultural antibiotic use are key contributing factors. The emergence of these highly resistant strains poses a significant threat, leading to increased morbidity, mortality, and healthcare costs in Ethiopia. The authors highlight the urgent need for national surveillance, genomic detection of resistance mechanisms, and strengthened antimicrobial stewardship programs to combat this escalating crisis and inform future research and policy development.

## 1. Introduction

The global rise of antimicrobial resistance (AMR) poses a significant threat to public health, undermining decades of progress in infectious disease management [1]. The most alarming developments within this crisis are the emergence of extensively drug-resistant (XDR) bacterial strains, which are non-susceptibility to at least one agent in all but two or fewer antimicrobial categories, and pan drug-resistant (PDR) strains, defined as isolates resistant to all available antimicrobial agents [2]. Studies conducted in Ethiopia reveal a concerning rise in antimicrobial resistance, with a notable prevalence of multidrug-resistant (MDR), XDR, and even PDR bacteria. The common bacteria reported were *Klebsiella pneumoniae*, *Acinetobacter baumannii*, *Staphylococcus aureus, Pseudomonas aeruginosa,* and *Escherichia coli*, which exhibit increasing resistance to a broad spectrum of antibiotics, including carbapenems [3–5].

An increased burden of antibiotic-resistant bacteria is related to a complex interplay of resistance mechanisms. The production of enzymes, such as carbapenemases and extended-spectrum beta-lactamases, which inactivate a broad range of antibiotics, alterations in bacterial cell membrane permeability, the action of efflux pumps, and the acquisition of resistance genes through horizontal gene transfer, often facilitated by plasmids, further contributes to the rapid dissemination of resistance [6]. Research indicates that these resistant strains are particularly prevalent in hospital settings, especially in intensive care units, and also within environmental samples such as those taken from water sources and waste disposal sites [7,8].

The issue of extensive drug resistance in Ethiopia extends to *Mycobacterium tuberculosis* (MTB), the causative agent of tuberculosis (TB). Ethiopia is recognized as a country with a high burden of both MDR and rifampicin-resistant TB [9]. The definition of pre-extensively drug-resistant TB (Pre-XDR-TB) refers to MDR-TB with resistance to any fluoroquinolone, whereas extensively drug-resistant TB (XDR-TB) is defined as MDR-TB with additional resistance to any fluoroquinolone and at least one Group A drug [10].

While existing research highlights the growing prevalence of MDR bacteria within the country, the progression towards XDR and PDR represents a critical public health problem. It is valuable to determine the current state of knowledge regarding the emergence and dissemination of XDR and PDR bacterial strains in Ethiopia. Therefore, this review focused on the documented cases of XDR and PDR bacterial infections, including Pre-XDR and XDR-TB,the potential factors contributing to their emergence, the current targets for AMR,their implications for public health, and insights for future research in Ethiopia.

## 2. Methodology

### 2.1. Study design

This scoping review of the literature was conducted according to the Preferred Reporting Items for Systematic Reviews and Meta-Analyses Extension for Scoping Reviews (PRISMA-ScR) guidelines [11] (S1 File). The study protocol was registered in the International Prospective Register of Systematic Reviews (PROSPERO), with identification number CRD420251084786 and link https://www.crd.york.ac.uk/PROSPERO/view/CRD420251084786.

### 2.2. Research questions

- What is the current state of XDR and PDR Gram-positive and Gram-negative bacterial infections?
- Are Pre-XDR and XDR-TB existing in the country of high TB burden?
- What are the possible contributing factors for the emergence of XDR and PDR bacteria in the Ethiopian context?
- What are current advancements and future perspectives forfighting AMR in the country?
- What will be their implications for Ethiopian public health for the implementation of preventive strategies and future research?

### 2.3. Literature search strategy

This scoping review used the Population–Concept–Context (PCC) paradigms to determine the suitability of the studies. It included studies on any patient population (“Population”) in Ethiopia (“Context”) that reported on XDR or PDR bacteria (including Pre-XDR/XDR-TB) as the main outcomes (“Concept”). The search included all available articles published; the last search was performed between March 10 and 20, 2025. The following electronic databases were used: PubMed/Medline, Scopus, EMBASE, Google Scholar, Hinari, Web of Science, Science Direct, Cochrane Library, and African Journals Online. The search terms were used alone or in combination with Boolean operators such as “OR” or “AND”. An example of a PubMed search strategy used was as follows: ((((((((antimicrobial resistance) OR (pre-extensively drug-resistant)) OR (extensively drug-resistant)) OR (pandrug-resistant)) OR (XDR)) OR (Pre-XDR)) OR (PDR)) AND (((((((((bacteria) OR (organism)) OR (infection*)) OR (*Mycobacterium*)) OR (*Mycobacterium tuberculosis*)) OR (*M. tuberculosis*)) OR (MTB)) OR (tuberculosis)) OR (TB))) AND (Ethiopia). A manual search of the references of the included studies and other reviews was conducted. The articles retrieved were imported into EndNote X9 bibliographic software manager (Clarivate Analytics, Philadelphia, PA, USA).

### 2.4. Study selection and eligibility criteria

The titles and abstracts of the studies were screened by four authors (MA, MT, WTA, and AA) independently. The full-text articles were then assessed for eligibility, and any disagreements between the authors were resolved through discussion with the fifth author (SB). Original research articles published in English that reported XDR and PDR, Pre-XDR-TB, and XDR-TB, conducted in Ethiopia only, and without limit on the study period and publication year were eligible for the study. Additionally, primary studies on clinical isolates were included except one study from environmental isolates.Case reports, communications, letters to editors, opinions, reviews, and meta-analysis studies were excluded.

### 2.5. Data extraction and synthesis

Data from individual studies were extracted using the designed extraction tool in Microsoft Excel 2019 (Microsoft Corp., Redmond, WA, USA) by four authors (MA, MT, AA, and GG). The type of information collected from eligible studies was author name, year of publication, area in which the study was conducted, study period, sample size, source of specimen,number of bacterial isolates, number of XDR/PDR bacteria, type of bacterial isolate, number of MTB/MDR-TB, Pre-XDR-TB, and XDR-TB.Because of the nature of the study design (scoping review), data were synthesized manually without using software. The overall prevalence was calculated using the following formula:

$\text{Prevalence}=\frac{\text{Sum of XDR or PDR bacteria}}{\text{Total number of bacteria}}*\text{ 100}\%$ and $\frac{\text{Pre}-\text{XDR or XDR}-\text{TB}}{\text{Total TB cases}}\;*\text{ 100}\%$

The minimum and maximum prevalence from studies was taken as a range value. Descriptive statistics were used to summarize the individual study’s findings.The results were presented in tables and figures.

## 3. Results

### 3.1. Literature search results

**Fig 1:**
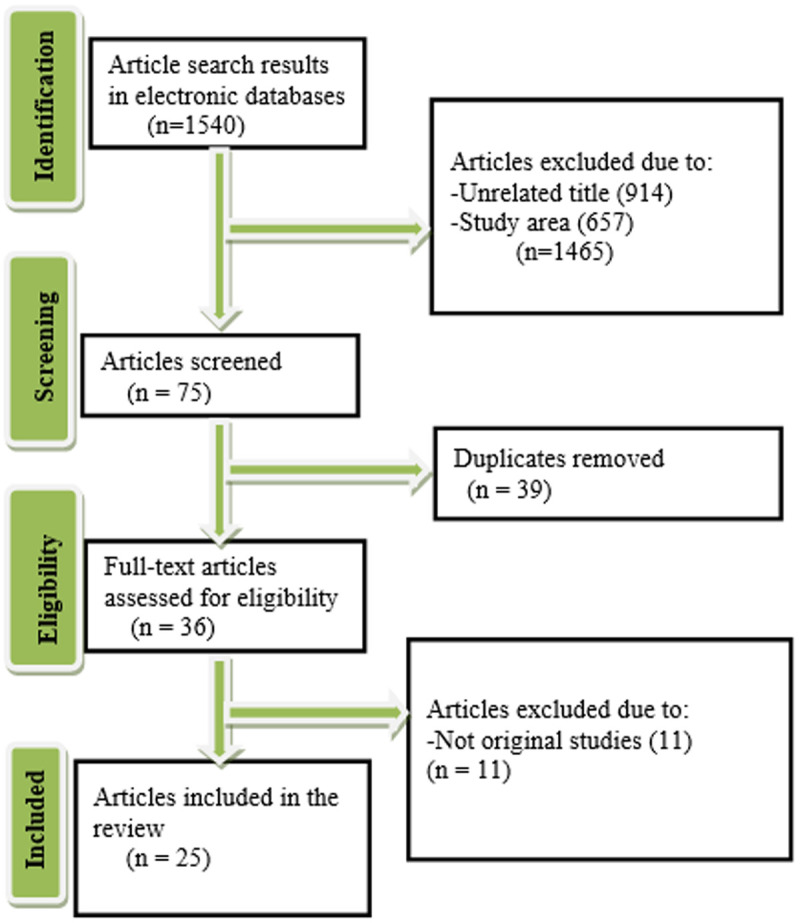
Flow diagram describing the selection of studies for the review.

In this review, 1540 potentially relevant articles were identified. Depending on the evaluation of the eligibility criteria, 25full-text articles were included in the review [12–37] (Fig 1).

### 3.2. Study demographics

Twenty-five studies (8 for TB and 17 for other bacteria) published between 2010 and 2025 were included in this review. Among seventeen studies, most of the single-centered studies were conducted in Addis Ababa (n = 8), followed by Bahir Dar (n = 2), one study each for Harar, Sidama, Jimma, and Arba Minch. Multi-center studies were also conducted; such as one each for (Addis Ababa, Harar, Jimma, and Hawassa), (Addis Ababa, Jimma, and Hawassa), and (Addis Ababa and Adama) (Table 1). On the other hand, eight studies were included for TB (3 from Addis Ababa,1 each from Amhara, Tigray, and Oromia regions, 2 from multiple regions of Ethiopia) (Table 2).

**Table 1:** Summary of studies reported XDR and PDR bacteria strains in Ethiopia.

Author (reference)	Study area	Study period	Sample size	Bacterial isolates	XDR (%)	PDR (%)	*Klebsiella spp.* (XDR, PDR)	*E. coli* (XDR, PDR)	*P. aeruginosa* (XDR, PDR)	*Acinetobacter spp.* (XDR, PDR)	Other least common bacteria
Gebremedhin et al, 2024 [19]	Addis Ababa & Adama	2022-23	688	151	43 (31.2)	–	–	–	–	–	*S. aureus,* CONS*, Enterococcus, Citrobacter spp., Enterobacter spp., Proteus spp., S. enterica sub. enterica, M. morganii, Providencia spp.,* and *Serratia spp.*
Gadisa et al, 2024a [26]	Addis Ababa, Harar, Jimma, & Hawassa	2021-23	5613	609	148 (24.3)	66 (10.9)	609 (NA, 66)	–	–	–	
Gadisa et al, 2024b [25]	Addis Ababa, Jimma, & Hawassa	2021-2023	3706	530	137 (25.9)	60 (11.4)	530 (137, 60)	–	–	–	
Erkihun et al, 2024 [14]	Bahir Dar	2020-22	2073	1258	405 (32.2)	183 (14.5)	391 (129, 101)	282 (76, 62)	140 (38, 6)	89 (9, 7)	
Addis et al, 2021b [22]	Addis Ababa	2021	NA	110	10 (9.0)	2 (1.8)	–	–	11 (1, NA)	–	
Geta & Kibret, 2022 [12]	Bahir Dar	2020-21	126	411	35 (8.5)	20 (4.9)	18 (7, 4)	82 (12, 9)	–	–	
Seid et al, 2025 [24]	Arba Minch	2019-22	207	176	10 (5.7)	–	34 (4, NA)	–	–	6 (3, NA)	
Abdeta et al, 2021 [28]	Addis Ababa	2019-20	1337	429	33 (7.7)	–	109 (3, NA)	–	36 (1, NA)	74 (24, NA)	
Beshah et al, 2022 [13]	Addis Ababa	2018-19	1486	427	75 (32.2)	17 (7.3)	75 (NA, 2)	38 (NA, 1)	–	47 (NA, 13)	
Beyene et al, 2019 [18]	Addis Ababa	2017-18	947	238	21 (8.8)	2 (0.8)	72 (8, 2)	144 (26, NA)	–	–	
Addis et al, 2021a [20]	Addis Ababa	2017-18	1012	319	66 (20.7)	11 (3.5)	30 (12, NA)	168 (30, 4)		12 (7, 3)	
Bitew & Tsige, 2020 [15]	Addis Ababa	2016-18	NA	440	151 (34.3)	35 (7.9)	56 (21, 8)	339 (121, 22)		–	
Bitew, 2019 [16]	Addis Ababa	2016-17	996	135	18 (13.3)	14 (10.4)	–	–	78 (7, 4)	47 (11, 10)	
Bitew et al, 2018 [37]	Addis Ababa	2016-17	422	64	27 (42.2)	2 (3.1)	13 (11, NA)	4 (1, NA)	4 (1, NA)	7 (4, NA)	
Mekonnen et al, 2023 [21]	Harar	2021	332	86	10 (14.7)	2 (2.3)	13 (1, 1)	28 (8, 1)	–	–	
Alemayehu et al, 2021 [23]	Sidama	2020	103	111	48 (43.2)	2 (1.8)	–	–	–	–	
Gashaw et al, 2018 [27]	Jimma	2016	118	126	52 (41.3)	24 (19.1)	30 (13, 2)	31 (10, 7)	9 (4, 3)	2 (1, 1)	

XDR; Extensively drug-resistant, PDR; Pandrug-resistant, CONS; Coagulase-negative *Staphylococcus*, *Spp.;* species*,* NA;Not Available.

**Table 2:** Summary of studies reported Pre-XDR and PDR-TB in Ethiopia.

Author (reference)	Study area	Study period	Sample size	Pre-XDR-TB (%)	XDR-TB (%)
Diriba et al, 2025 [29]	Addis Ababa	2022-24	468 (MDR-TB)	11 (2.4)	5 (1.1)
Diriba et al, 2022 [30]	Addis Ababa	2019-22	644 (MDR-TB)	19 (3.0)	–
Dagne et al, 2021 [33]	Multiple region	2019-20	160 (TB)	8 (5.0)	1 (0.6)
Yenew et al, 2024 [34]	Multiple region	2019-20	19 (MDR-TB)	1 (5.3)	1 (5.3)
Welekidan et al, 2020 [35]	Tigray region	2018-19	38 (MDR-TB)	2 (5.3)	–
Bedru et al, 2021 [32]	Oromia region	2017-18	30 (MDR-TB)	1 (3.3)	3 (10.0)
Shibabaw et al, 2020 [31]	Amhara region	2016-18	174 (MDR-TB)	10 (5.7)	1 (0.6)
Agonafir et al, 2010 [36]	Addis Ababa	2005-6	46 (MDR-TB)	–	2 (4.4)

MDR-TB; Multidrug-resistant Tuberculosis, Pre-XDR-TB; Pre- extensively drug-resistant Tuberculosis, XDR-TB; Extensively drug-resistant Tuberculosis.

### 3.3. Extensively drug-resistant bacterial isolates

Among a total of 5620 bacterial isolates identified,1289 were XDR (22.9%; 1289/5620), with the prevalence ranging from 5.7% to 43.2%. The most common XDR bacteria identified were *Klebsiella* species; 26.7% (2.8%-84.6%), followed by *E. coli*; 26.4% (14.6%-35.7%), *Acinetobacter* species*;* 24.9%(10.1%-58.3%), and *P. aeruginosa;* 18.7% (2.8%-44.4%) (Table 1). The least common isolates reported were *S. aureus,* Coagulase-negative *Staphylococcus, Enterococcus, Citrobacter* species*., Enterobacter* species*, Proteus* species*., S. enterica sub. enterica, M. morganii, Providencia* species*,* and *Serratia* species (S2 File). The presence of Gram-negative XDR bacteria across the studies within different sources of specimens and environments represents a significant concern in community and hospital settings.

### 3.4. Pandrug-resistant bacterial isolates

According to recent studies in Ethiopia, a total of 440 bacterial isolates were PDR (9.1%; 440/4864 total isolates), with its prevalence in individual studies ranging from 0.8% to 19.1%. The most frequently identified PDR bacteria were *Acinetobacter* species*;* 17.3% (7.9%-50.0%), followed by *Klebsiella* species*;* 13.7%(2.7%-25.8%), *E. coli*; 10.2% (2.4%-22.6%), and *P. aeruginosa;* 5.7%(4.3%-33.3%) (Table 1).While seemingly less prevalent than XDR, the identification of PDR bacteria in multiple studies across Ethiopia signifies a critical and advanced stage in antibiotic resistance within the country.

### 3.5. Pre-extensively and extensively drug-resistant tuberculosis

A total of eight studies [29–36] comprising, 1419 MDR-TB and 160 TB confirmed cases were included. The varying prevalence rates of Pre-XDR-TB; 3.4% (2.4%-5.7%) and XDR-TB; 1.5% (0.6%-10.0%) have been reported. The emergence of XDR in MTB alongside other bacterial species given a broader underlying issue in Ethiopia (**Table 2**).

## 4. Discussion

This review is the first to describe the findings of original research articles on the currently emerging XDR and PDR bacterial strains in Ethiopia. The comprehensive summary of findings from different regions of Ethiopia showed an overall prevalence of XDR (22.9%; 1289 XDR bacteria divided by 5620 total bacterial isolates) and PDR (9.1%; 440 PDR bacteria divided by 4864 total bacterial isolates), with highly prevalent Gram-negative bacteria. In a systemic review of the current epidemiology and prognosis of PDR Gram-negative bacteria, a total of 526 PDR isolates were reported with *P. aeruginosa* (33.3%), *A. baumannii* (32.7%) and *K. pneumoniae* (23.8%). The majority of PDR strains were isolated from intensive care units, with the potential to cause hospital outbreaks and dissemination between hospitals and long-term facilities. Pan-drug-resistant infections were associated with excess mortality, mounting up to 71.0%, and were independently high regardless of the infection source [38]. Thus, this review described the burden of XDR and PDR, and their mechanism of resistance on frequently reported bacteria such as *Klebsiella* species*, Acinetobacter* species*, E. coli*, and *P. aeruginosa* from an Ethiopian perspective.

### 4.1. Emergence of XDR and PDR *Klebsiella* species

Antibiotic resistance usually emerges among Gram-negative bacilli, especially Entero bacteriaceae because of their prevalence in hospital settings and the spectrum of resistance to antibiotics by *K. pneumoniae* is gradually increasing. This review found that *Klebsiella* species were the most commonly identified bacteria with XDR; 26.8% and PDR; 13.7%.This finding is comparable with a study by Usman et al, which reported the XDR (31.3%) and PDR (9.4%) prevalence of *K.pneumoniae* [39]. A study in India also reported an 18.0% prevalence of XDR *Klebsiella* species [40].In recent years, the AMR problem of *Klebsiella* species has become increasingly severe. In the case of *K. pneumoniae*, genes like *bla-KPC* and *bla-NDM* confer resistance to β-lactams including carbapenems; *mcr-1* leads to colist in resistance; *K.oxytoca, bla-CTX-M* causes resistance to multiple β-lactams, and *aac(3)-II* results in aminoglycoside resistance; *K. granulomatis* may have *tet* genes, making it resistant to tetracycline [41]. Since the virulence potential of this pathogen is increasing, further research should be conducted on hypervirulent strains to compare virulence with resistance patterns for the development of treatment targets.

### 4.2. Emergence of XDR and PDR *Acinetobacter* species

Globally,*Acinetobacter* species have been a leading cause of nosocomial infections, causing significant morbidity and mortality.In this review,the burden of their XDR and PDR patterns showed 24.9% and 17.3%, respectively. A five-year antimicrobial resistance trend analysis showed increasing carbapenem non-susceptible and MDR rates in *Acinetobacter* species*,* with 56.7% and 71.6%, respectively [42]. A study in Bangladesh found moderate to high levels of resistance against aminoglycosides (45–53%), cephalosporins (28–45%), fluoroquinolones (28–39%), and carbapenems (17–19%), as well as XDR (13.64%) and PDR (2.3%) isolates of *Acinetobacter* species [43]. According to virulence-associated phenotypic assays, XDR isolates were more virulent to *G. mellonella* larvae, had a better capacity for iron uptake, and produced more capsules. Furthermore, virulence genes (*tonB, hemO, abaI,* and *ptk*) were more prevalent in XDR isolates, whereas *pld* and *ompA* genes were more prevalent in non-MDR isolates [44]. The most important features of *A*. *baumannii* are its ability to persist in hospital settings and rapidly develop resistance to a wide variety of antibiotics. Compared with other bacteria, *A.baumannii* has a highly sophisticated resistance mechanism including various antimicrobial-inactivating enzymes, efflux pump over expression, alterations in antibiotic target location, and outer membrane protein permeability [45].

### 4.3. Emergence of XDR and PDR *E. coli*

The prevalence of XDR and PDR *E. coli* isolates was 26.3% and 10.2%, respectively. This finding is supported by a study in Iraq that reported XDR strains (25.4%) [46]. The key resistance mechanisms in *E. coli* include efflux pumps and porin mutations that mediate resistance to a broad spectrum of antibiotics, biofilm formation, persister cell formation, the activation of stress response systems, to withstand antibiotic pressure, and the acquisition of resistance genes through horizontal gene transfer, facilitated by mobile genetic elements such as plasmids and transposons [47]. The presence of transferable plasmids is responsible for the XDR phenotype of E. coli W60; NDM-5 confers high resistance to β-lactam/BLI combinations; co-expression of *ble*-MBL enhances the resistance caused by NDM-5; and the secretion and function of TEM type β-lactamases depend on their signal peptides [48].

### 4.4. Emergence of XDR and PDR *P. aeruginosa*

*P. aeruginosa* is one of the most common antibiotic-resistant bacteria causing nosocomial infections, especially in burn units and patients with cystic fibrosis [49,50]. The combined report of this review showed an XDR; 18.7% (2.8%-44.4%) and PDR; 5.7% (4.3%-33.3%) prevalence of *P. aeruginosa.* The antibiotic resistance profile of *P. aeruginosa* in intensive care units revealed that 50% were MDR, and 2.3% were XDR phenotype [51]. Another study on burn patients showed an 87.5% XDR prevalence [52]. Major resistance mechanisms are intrinsic, acquired, and adaptive, which include biofilm-mediated resistance and the formation of multidrug-tolerant persister cells [53,54]. The intrinsic resistance occurs through restricted outer membrane permeability, the presence of efflux systems, and the production of antibiotic-inactivating enzymes. Whereas acquired resistance occurs either through horizontal gene transfer (acquisition of aminoglycoside-modifying enzymes and β-lactamases) or mutational events that result in the overexpression of efflux pumps or β-lactamases or the decreased expression or modification of target sites and porins [55].

### 4.5. The Burden of Pre-XDR and XDR-TB in Ethiopia

According to the studies report, there were varying prevalence rates of Pre-XDR-TB; 3.4% (2.4%-5.7%) and XDR-TB; 1.5% (0.6%-10.0%). Compared to our review finding, a higher prevalence of Pre-XDR and XDR-TB was reported from India, with 55.9% and 4.9%, respectively [56]. A systematic review and meta-analysis in Latin America and the Caribbean showed that the pooled prevalence of pre-XDR-TB was 10.0%, being higher in Brazil (16.0%) and Peru (13.0%), whereas the pooled prevalence of XDR-TB in was 5.0%, being higher in Cuba and Peru, 6.0% each [57].Another study from Bangladesh reported 16.18% of Pre-XDR-TB cases, with 81.82% fluoroquinolone-resistant Pre-XDR-TB and 18.18% of second-line injectable agent-resistant Pre-XDR-TB [58]. Moreover, Daniel et al reported a Pre-XDR TB prevalence rate of 16.7%, with 80.0% resistance to ofloxacin and 20.0% resistance to Kanamycin [59]. A study determined the role of DNA gyrase mutations in Pre-XDR AND XDR-TB clinical isolates revealed that all Pre-XDR-TB and XDR-TB isolates carried at least one mutation within the quinolone resistance-determining region of DNA gyrase [60].Thisvariation could be due to the meta-analysis nature of the study based on diverse patient populations in various settings, geographic differences, methods of detection, and epidemiological factors that contribute to drug resistance. This review finding provides insight into the current issue of Pre-XDR and XDR-TB strains in different regions of Ethiopia, which requires strengthening the national drug-resistance surveillance and TB programs, and continued efforts in TB control and management.

## 5. Factors contributing to XDR and PDR in the Ethiopian context

Antibiotic usage patterns are a major factor in the emergence and spread of XDR and PDR bacteria in Ethiopia. The inappropriate use of antimicrobials includes the overuse of antibiotics, their misuse for non-bacterial infections, their availability over the counter without a prescription, and the common practice of empirical prescribing based on clinical syndromes rather than definitive microbiological diagnosis [61]. Additionally, incomplete courses of antibiotic therapy and prolonged treatment durations also contribute to the selection and propagation of antibiotic-resistant strains [62]. A study on antibiotic consumption and prescribing patterns in Ethiopia reveals high usage of certain antibiotics like doxycycline, amoxicillin, and ciprofloxacin, alongside reports of irrational prescribing practices involving incorrect doses, frequencies, and durations [63]. This resulted in a significant selective pressure, favoring the survival and proliferation of bacteria that have developed resistance mechanisms, ultimately leading to the emergence of XDR and PDR strains.

Inadequate sanitation and hygiene infrastructure, limited access to clean water, inadequate management of waste in both healthcare facilities and communities, and insufficient adherence to infection prevention and control guidelines all contribute to the dissemination of drug-resistant microorganisms [61].A study has documented that suboptimal water, sanitation, and hygiene facilities, as well as low rates of hand-hygiene compliance within healthcare settings, are risk factors for antibiotic-resistant infections [64]. Moreover, the presence of antibiotic-resistant bacteria and resistance genes in healthcare wastewater and municipal solid waste highlights the environmental dimension of this challenge [65].

The use of antibiotics in agriculture and animals is another recognized contributing factor to the rise of AMR in Ethiopia. The practice of administering antimicrobials to farm animals for prophylactic purposes and to promote growth can lead to the development of resistant bacteria in animals, which can then potentially transfer to humans through the food chain or environmental contamination [66]. This interconnectedness between human and animal health, often referred to as the “One Health” approach, underscores the need to consider antibiotic use across all sectors. While Ethiopia has established national action plans for AMR, challenges persist in ensuring their effective implementation, coordination among different stakeholders, and adequate allocation of financial resources [62].

## 6. Current solutions and future perspectives for tackling AMR in Ethiopia

Ethiopia has recognized the growing threat of AMR and has developed a National Action Plan for Antimicrobial Resistance (NAP-AMR) from 2021 to 2025 [67]. This plan adopts a “One Health” approach, acknowledging the interconnectedness of human, animal, and environmental health in the context of AMR. The challenges of AMR in Ethiopia require alternative treatment strategies beyond conventional antibiotics. Given the limitations of existing antibiotics, recent research into phage therapy, which utilizes bacteriophages to target and destroy specific bacteria, holds significant promise. A study by Hailemichael et alreported that virulent phages were active against 42% of MDR *A. baumannii*, 40% of both biofilm-producing and MDR *A. baumannii*, and 35.3% of the biofilm-producing isolates [68].The promising effect of the *Myoviridae*-like phages, *Podoviridae*, and *Siphoviridae* phages against drug-resistant pathogenic *E. coli* has raised the possibility of their use in the future treatment of *E. coli* infections [69]. Another study also suggested that phages (ΦJHS-PA1139 and ΦSMK-PA1139) have great potential to serve the dual purpose as surface coating agents for preventing MDR *P. aeruginosa* colonization in medical implants and as biofilm removal agents in implant-associated infections [70].

Additionally, investigating the potential of traditional Ethiopian medicinal practices, particularly those involving plant-derived compounds with antimicrobial properties, could yield valuable insights to hinder challenges associated with emerging antimicrobial resistance. An in-vitro experimental study conducted by Gadisa and Tadesse showed that the extracts obtained from *C.englerianum* and *E. depauperate* had potent antibacterial activity on MDR bacteria such as Methicillin-resistant *S. aureus*, *E. faecalis, E. coli*, and *K. pneumoniae* [71].Strengthening infection prevention and control measures within healthcare settings is paramount to minimize the spread of resistant bacteria. Moreover, improving the laboratory capacity in Ethiopia for proper identification of resistant bacteria and sensitivity testing of detection methods could be valuable. This will help to reduce the inappropriate use of antibiotics, which is a major contributor to the growing problem of antibiotic resistance.

## 7. Implications for public health and research

The emergence of XDR and PDR bacterial infections in Ethiopia has a significant impact on public health, causing high morbidity and mortality. Because of their resistance to most or all conventional antibiotics, the presence of XDR and PDR bacteria also presents a significant treatment challenge and severely limits therapeutic options The healthcare system in Ethiopia is facing a dwindling arsenal of effective antimicrobial agents to combat these life-threatening infections [72]. This scarcity of treatment choices can lead to treatment failures, prolonged periods of illness, and an increased reliance on potentially more harmful medications. Vulnerable populations such as newborns and immune-compromised patients within Ethiopia are at a higher risk of drug-resistant bacterial infections. Studies have specifically highlighted the high prevalence of MDR bacteria among HIV-positive individuals [73] and the significant impact of drug-resistant bacteria among hospitalized neonates for clinical bloodstream infections [74].

The genomic epidemiology of β-lactamases, carbapenemases, colistin-resistance, and other resistance genes in Ethiopia reveals a concerning landscape of rapidly evolving AMR, significantly compromising the effectiveness of last-resort antibiotics. This detailed genetic understanding highlights the prevalence and diverse types of carbapenemase genes, particularly *bla-NDM* variants, and their presence across various clinically relevant bacteria like *K. pneumoniae*, *A. baumannii*, and *E. coli*, often co-harboring multiple resistance mechanisms [75,76]. It signals a heightened risk of untreatable infections, leading to increased patient morbidity, mortality, prolonged hospital stays, and escalating healthcare costs in an already resource-constrained setting. Identifying the specific mobile genetic elements (like plasmids) carrying these resistance genes underscores the high potential for horizontal gene transfer, facilitating their rapid spread not only within healthcare settings but also across human, animal, and environmental reservoirs, demanding a robust “One Health” approach to surveillance and intervention strategies. This genomic insight is vital for informing targeted infection control, guiding antimicrobial stewardship programs, and accelerating the development of novel diagnostics and therapeutic approaches to combat this escalating public health crisis in Ethiopia.It is important to strengthen laboratory capacity and surveillance networks in line with WHO recommendations for AMR containment.

The identification of XDR-TB in Ethiopia is a challenge to effective TB management, which limits treatment options, leading to higher rates of treatment failure, relapse, and mortality. This poses a significant burden on Ethiopia’s healthcare system, which requires an increased cost of specialized drugs, prolonged treatment durations, and the need for more intensive patient monitoring and support, all within a resource-limited setting. Furthermore, the presence of XDR-TB resulted in the risk of widespread transmission, particularly in crowded urban areas and within vulnerable populations. Strengthening rapid and accurate drug susceptibility testing, ensuring strict adherence to directly observed treatment for all TB patients, innovating new and effective anti-TB drugs, and implementing comprehensive contact tracing and investigation to identify and manage new cases swiftly are strategies to curb its spread.

## 8. Strengths and limitations

Although the researchers provided valuable input regarding the recently emerging XDR and PDR bacterial strains, it is important to note that the true burden of XDR and PDR bacteria in Ethiopia might be underestimated due to inherent challenges in their phenotypic detection (disk-diffusion method) and reporting variation. As highlighted by Global Antibiotic Research and Development Partnership (GARDP) Revive, the prevalence of PDR bacteria is difficult to accurately assess because isolates are often not tested against all possible antibiotics. This limitation in comprehensive testing, potentially due to resource constraints, suggests that the reported prevalence rates may not fully reflect the actual extent of XDR and PDR in the country.

## 9. Conclusion

In Ethiopia, the emergence of XDR and PDR bacteria represents a significant and growing threat to public health. These highly resistant bacteria pose a serious challenge to the healthcare system, leading to increased morbidity, mortality, prolonged hospitalizations, and high healthcare costs. Sustained and intensified implementation of Ethiopia’s NAP-AMR, with adequate funding and strong multisectoral coordination, is crucial. Strengthening surveillance systems to better track the emergence and spread of resistant bacteria, promoting antimicrobial stewardship programs to optimize antibiotic use, and enhancing infection prevention and control measures are mandatory.

### 9.1. Significance of the review

This review provides evidence for recently emerging XDR and PDR bacterial strains, as well as Pre-XDR and XDR-TB in Ethiopia. Additionally, it explored the significant contributors of resistance, recent advancements in the treatment of drug-resistant bacteria in Ethiopia, and their implications for public health. It highlights the need for urgent nationwide surveillance and antimicrobial stewardship programs. The findings also serve as baseline data for future research and healthcare policy development at the national level.

## Supporting information

S1 File PRISMA-ScR Checklist. (DOCX)

S2 File The detailed information of included articles for this review. (XLSX)

S3 File Abbreviations. (DOCX)

## References

[1] Aijaz M, Ahmad M, Ansari MA, Ahmad S. Antimicrobial resistance in a globalized world: current challenges and future perspectives. J Pharm Drug Deliv. 2023;1(1):7–22.

[2] Magiorakos A-P, Srinivasan A, Carey RB, Carmeli Y, Falagas ME, Giske CG, et al. Multidrug-resistant, extensively drug-resistant and pandrug-resistant bacteria: an international expert proposal for interim standard definitions for acquired resistance. Clin Microbiol Infect. 2012;18(3):268–81. doi: 10.1111/j.1469-0691.2011.03570.x 21793988

[3] Alemayehu T. Prevalence of multidrug-resistant bacteria in Ethiopia: a systematic review and meta-analysis. J Glob Antimicrob Resist. 2021;26:133–9. doi: 10.1016/j.jgar.2021.05.017 34129993

[4] Abayneh M, Zeynudin A, Tamrat R, Tadesse M, Tamirat A. Drug resistance and extended-spectrum β-lactamase (ESBLs) - producing Enterobacteriaceae, Acinetobacter and Pseudomonas species from the views of one-health approach in Ethiopia: a systematic review and meta-analysis. One Health Outlook. 2023;5(1):12. doi: 10.1186/s42522-023-00088-z 37697359 PMC10496308

[5] Berhe DF, Beyene GT, Seyoum B, Gebre M, Haile K, Tsegaye M, et al. Prevalence of antimicrobial resistance and its clinical implications in Ethiopia: a systematic review. Antimicrob Resist Infect Control. 2021;10(1):168. 34861894 10.1186/s13756-021-00965-0 PMC8642948

[6] Zhang F, Cheng W. The Mechanism of Bacterial Resistance and Potential Bacteriostatic Strategies. Antibiotics (Basel). 2022;11(9):1215. doi: 10.3390/antibiotics11091215 36139994 PMC9495013

[7] Aleem M, Azeem AR, Rahmatullah S, Vohra S, Nasir S, Andleeb S. Prevalence of Bacteria and Antimicrobial Resistance Genes in Hospital Water and Surfaces. Cureus. 2021;13(10):e18738. doi: 10.7759/cureus.18738 34790487 PMC8587521

[8] Osman A-H, Darkwah S, Kotey FCN, Odoom A, Hotor P, Dayie NTKD, et al. Reservoirs of Nosocomial Pathogens in Intensive Care Units: A Systematic Review. Environ Health Insights. 2024;18:11786302241243239. doi: 10.1177/11786302241243239 38828046 PMC11141231

[9] Demelash M, Nibret E, Hailegebriel T, Minichil Z, Mekonnen D. Prevalence of rifampicin resistant pulmonary tuberculosis using geneXpert assay in Ethiopia, a systematic review and meta-analysis. Heliyon. 2023;9(9):e19554. doi: 10.1016/j.heliyon.2023.e19554 37809604 PMC10558782

[10] Organization WH. Global tuberculosis report 2021: supplementary material. World Health Organization. 2022.

[11] Page OJBJSM. Preferred reporting items for systematic reviews and meta-analyses extension for scoping reviews (PRISMA-ScR) checklist. OJBJSM. 2024;1001:58.

[12] Geta K, Kibret M. Antibiotic Resistance Profiles of Bacteria Isolated from Hotspot Environments in Bahir Dar City, Northwestern Ethiopia. J Multidiscip Healthc. 2022;15:1403–14. doi: 10.2147/JMDH.S364324 35785260 PMC9242431

[13] Beshah D, Desta A, Belay G, Abebe T, Gebreselasie S, Sisay Tessema T. Antimicrobial Resistance and Associated Risk Factors of Gram-Negative Bacterial Bloodstream Infections in Tikur Anbessa Specialized Hospital, Addis Ababa. Infect Drug Resist. 2022;15:5043–59. doi: 10.2147/IDR.S371654 36068835 PMC9441145

[14] Erkihun M, Assefa A, Legese B, Almaw A, Berhan A, Getie B, et al. Epidemiology and antimicrobial resistance profiles of bacterial isolates from clinical specimens at Felege Hiwot Comprehensive Specialized Hospital in Ethiopia: retrospective study. 2024;3(4):405–21.

[15] Bitew A, Tsige E. High prevalence of multidrug-resistant and extended-spectrum β-lactamase-producing Enterobacteriaceae: A cross-sectional study at Arsho Advanced Medical Laboratory, Addis Ababa, Ethiopia. J Trop Med. 2020;2020:6167234. 32411256 10.1155/2020/6167234 PMC7210541

[16] Bitew A. High prevalence of multi-drug resistance and extended spectrum beta lactamase production in non-fermenting gram-negative bacilli in Ethiopia. Infect Dis (Auckl). 2019;12:1178633719884951. 31723320 10.1177/1178633719884951 PMC6836305

[17] Bitew AJEM. Multi-drug resistance profile of bacteria isolated from blood stream infection at Tikur anbessa specialized hospital, Addis Ababa, Ethiopia. Ethiopian Journal of Health Sciences. 2018;14:119–26.

[18] Beyene D, Bitew A, Fantew S, Mihret A, Evans M. Multidrug-resistant profile and prevalence of extended spectrum β-lactamase and carbapenemase production in fermentative Gram-negative bacilli recovered from patients and specimens referred to National Reference Laboratory, Addis Ababa, Ethiopia. PLoS One. 2019;14(9):e0222911. doi: 10.1371/journal.pone.0222911 31553773 PMC6760794

[19] Gebremedhin KB, Yisma E, Alemayehu H, Medhin G, Belay G, Bopegamage S, et al. Urinary tract infection among people living with human immunodeficiency virus attending selected hospitals in Addis Ababa and Adama, central Ethiopia. Front Public Health. 2024;12:1394842. doi: 10.3389/fpubh.2024.1394842 39296834 PMC11408745

[20] Addis T, Mekonnen Y, Ayenew Z, Fentaw S, Biazin H. Bacterial uropathogens and burden of antimicrobial resistance pattern in urine specimens referred to Ethiopian Public Health Institute. PLoS One. 2021;16(11):e0259602. doi: 10.1371/journal.pone.0259602 34767605 PMC8589166

[21] Mekonnen S, Tesfa T, Shume T, Tebeje F, Urgesa K, Weldegebreal F. Bacterial profile, their antibiotic susceptibility pattern, and associated factors of urinary tract infections in children at Hiwot Fana Specialized University Hospital, Eastern Ethiopia. PLoS One. 2023;18(4):e0283637. doi: 10.1371/journal.pone.0283637 37018232 PMC10075463

[22] Addis T, Araya S, Desta K. Occurrence of Multiple, Extensive and Pan Drug-Resistant Pseudomonas aeruginosa and Carbapenemase Production from Presumptive Isolates Stored in a Biobank at Ethiopian Public Health Institute. Infect Drug Resist. 2021;14:3609–18. doi: 10.2147/IDR.S327652 34511952 PMC8427834

[23] Alemayehu T, Asnake S, Tadesse B, Azerefegn E, Mitiku E, Agegnehu A, et al. Phenotypic Detection of Carbapenem-Resistant Gram-Negative Bacilli from a Clinical Specimen in Sidama, Ethiopia: A Cross-Sectional Study. Infect Drug Resist. 2021;14:369–80. doi: 10.2147/IDR.S289763 33564245 PMC7866937

[24] Seid M, Bayou B, Aklilu A, Tadesse D, Manilal A, Zakir A, et al. Antimicrobial resistance patterns of WHO priority pathogens at general hospital in Southern Ethiopia during the COVID-19 pandemic, with particular reference to ESKAPE-group isolates of surgical site infections. BMC Microbiol. 2025;25(1):84. doi: 10.1186/s12866-025-03783-1 39987036 PMC11846185

[25] Gadisa E, Egyir B, Fekede E, Adu B, Danso J, Oclu A, et al. Epidemiology, antimicrobial resistance profile, associated risk factors and management of carbapenem resistant Klebsiella pneumoniae in children under 5 with suspected sepsis in Ethiopia. BMC Infect Dis. 2024;24(1):1458. doi: 10.1186/s12879-024-10366-4 39716087 PMC11665230

[26] Gadisa E, Egyir B, Adu B, Ahmed H, Disasa G, Tessema TS. Epidemiology, antimicrobial resistance profile and management of carbapenem-resistant Klebsiella pneumoniae among mothers with suspected sepsis in Ethiopia. Ann Clin Microbiol Antimicrob. 2024;23(1):85. doi: 10.1186/s12941-024-00745-9 39322956 PMC11423506

[27] Gashaw M, Berhane M, Bekele S, Kibru G, Teshager L, Yilma Y, et al. Emergence of high drug resistant bacterial isolates from patients with health care associated infections at Jimma University medical center: a cross sectional study. Antimicrob Resist Infect Control. 2018;7:138. doi: 10.1186/s13756-018-0431-0 30479751 PMC6245755

[28] Abdeta A, Bitew A, Fentaw S, Tsige E, Assefa D, Lejisa T, et al. Phenotypic characterization of carbapenem non-susceptible gram-negative bacilli isolated from clinical specimens. PLoS One. 2021;16(12):e0256556. doi: 10.1371/journal.pone.0256556 34855767 PMC8638961

[29] Diriba G, Alemu A, Yenew B, Ayano BZ, Hailu M, Buta B, et al. Second-line drug resistance among multidrug-resistant tuberculosis patients in Ethiopia: A laboratory-based surveillance. J Glob Antimicrob Resist. 2025;42:167–74. doi: 10.1016/j.jgar.2025.02.014 39988072

[30] Diriba G, Alemu A, Tola HH, Yenew B, Amare M, Eshetu K, et al. Pre-extensively drug-resistant tuberculosis among multidrug-resistant tuberculosis patients in Ethiopia: a laboratory-based surveillance study. IJID Reg. 2022;5:39–43. doi: 10.1016/j.ijregi.2022.08.012 36176268 PMC9513164

[31] Shibabaw A, Gelaw B, Gebreyes W, Robinson R, Wang S-H, Tessema B. The burden of pre-extensively and extensively drug-resistant tuberculosis among MDR-TB patients in the Amhara region, Ethiopia. PLoS One. 2020;15(2):e0229040. doi: 10.1371/journal.pone.0229040 32053661 PMC7018133

[32] Bedru H, Fikru M, Niguse W, Jemal A, Getinet G, Gobena A, et al. Drug Resistance Pattern of M. tuberculosis Complex in Oromia Region of Ethiopia. Infect Drug Resist. 2021;14:1679–89. 33976556 10.2147/IDR.S294559 PMC8106478

[33] Dagne B, Desta K, Fekade R, Amare M, Tadesse M, Diriba G, et al. The Epidemiology of first and second-line drug-resistance Mycobacterium tuberculosis complex common species: Evidence from selected TB treatment initiating centers in Ethiopia. PLoS One. 2021;16(1):e0245687. doi: 10.1371/journal.pone.0245687 33507946 PMC7842946

[34] Yenew B, Kebede A, Alemu A, Diriba G, Mehammed Z, Amare M, et al. Genotypic and phenotypic drug resistance patterns of Mycobacterium tuberculosis isolated from presumptive pulmonary tuberculosis patients in Ethiopia: A multicenter study. PLoS One. 2024;19(5):e0303460. 10.1371/journal.pone.0303460 PMC11098317 38753615

[35] Welekidan LN, Skjerve E, Dejene TA, Gebremichael MW, Brynildsrud O, Agdestein A, et al. Characteristics of pulmonary multidrug-resistant tuberculosis patients in Tigray Region, Ethiopia: A cross-sectional study. PLoS One. 2020;15(8):e0236362. doi: 10.1371/journal.pone.0236362 32797053 PMC7428183

[36] Agonafir M, Lemma E, Wolde-Meskel D, Goshu S, Santhanam A, Girmachew F, et al. Phenotypic and genotypic analysis of multidrug-resistant tuberculosis in Ethiopia. Int J Tuberc Lung Dis. 2010;14(10):1259–65. 20843416

[37] Bitew A. Multi-Drug Resistance Profile of Bacteria Isolated from Blood Stream Infection at Tikur Anbessa Specialized Hospital, Addis Ababa, Ethiopia. EC Microbiology. 2018;14:119–26.

[38] Karakonstantis S, Kritsotakis EI, Gikas AJ. Pandrug-resistant Gram-negative bacteria: a systematic review of current epidemiology, prognosis and treatment options. J JoAC. 2020;75(2):271–82. 10.1093/jac/dkz401 31586417

[39] Usman NI, Abdulwahab NM, Sulaiman MJ, Abdullahi S. Multidrug Resistance (MDR), Extensive Drug Resistance (XDR) and Pan Drug Resistance (PDR) Klebsiella Pneumoniae from Clinical Samples. 2022;3:42–50.

[40] Ghogale S, Pathak K. Occurrence of gram-negative bacterial pathogens that are multidrug-resistant (MDR), extensively drug-resistant (XDR), and pan-drug-resistant (PDR) in an Indian tertiary care hospital. 2023.

[41] Li J, Shi Y, Song X, Yin X, Liu H. Mechanisms of Antimicrobial Resistance in Klebsiella: Advances in Detection Methods and Clinical Implications. Infect Drug Resist. 2025;18:1339–54. doi: 10.2147/IDR.S509016 40092844 PMC11910031

[42] Ayenew Z, Tigabu E, Syoum E, Ebrahim S, Assefa D, Tsige E. Multidrug resistance pattern of Acinetobacter species isolated from clinical specimens referred to the Ethiopian Public Health Institute: 2014 to 2018 trend anaylsis. PLoS One. 2021;16(4):e0250896. doi: 10.1371/journal.pone.0250896 33914829 PMC8084144

[43] Asaduzzaman M, Nasrin N, Yeasmin T, Das SC, Islam S, Ahmed MJBPJ. Clinical evidence of multi-drug resistant, extensively drug resistant and pan-drug resistant Acinetobacter sp. in Bangladesh. Journal of Bangladesh Pharmaceutical Journal. 2024;27(1):67–72.

[44] Chen PK, Liu C-Y, Kuo H-Y, Lee Y-T, Liu Y-H, Zhang Y-Z, et al. Emergence of extensively-drug-resistant hypervirulent Acinetobacter baumannii isolated from patients with bacteraemia: bacterial phenotype and virulence analysis. Int J Antimicrob Agents. 2024;64(6):107358. doi: 10.1016/j.ijantimicag.2024.107358 39414173

[45] Shi J, Cheng J, Liu S, Zhu Y, Zhu M. Acinetobacter baumannii: an evolving and cunning opponent. Front Microbiol. 2024;15:1332108. doi: 10.3389/fmicb.2024.1332108 38318341 PMC10838990

[46] Jawad R, Alshami Z, Jiham H. Prevalence of colistin pan resistance among multidrug-resistant and extensively drug-resistant Escherichia coli O157: H7. 2024.

[47] Nasrollahian S, Graham JP, Halaji M. A review of the mechanisms that confer antibiotic resistance in pathotypes of E. coli. Front Cell Infect Microbiol. 2024;14:1387497. doi: 10.3389/fcimb.2024.1387497 38638826 PMC11024256

[48] Wang M, Wang W, Niu Y, Liu T, Li L, Zhang M, et al. A Clinical Extensively-Drug Resistant (XDR) Escherichia coli and Role of Its β-Lactamase Genes. Front Microbiol. 2020;11:590357. doi: 10.3389/fmicb.2020.590357 33362736 PMC7758502

[49] Verma U, Kulshreshtha S, Khatri PJI. MDR Pseudomonas aeruginosa in nosocomial infection: burden in ICU and burn units of a tertiary care hospital. 2018;7:1267–74.

[50] Bonyadi P, Saleh NT, Dehghani M, Yamini M, Amini K. Prevalence of antibiotic resistance of Pseudomonas aeruginosa in cystic fibrosis infection: A systematic review and meta-analysis. Microb Pathog. 2022;165:105461. doi: 10.1016/j.micpath.2022.105461 35240288

[51] Gill JS, Arora S, Khanna SP, Kumar KH. Prevalence of Multidrug-resistant, Extensively Drug-resistant, and Pandrug-resistant Pseudomonas aeruginosa from a Tertiary Level Intensive Care Unit. J Glob Infect Dis. 2016;8(4):155–9. doi: 10.4103/0974-777X.192962 27942195 PMC5126754

[52] Hosseininassab Nodoushan SA, Yadegari S, Moghim S, Isfahani BN, Fazeli H, Poursina F, et al. Distribution of the Strains of Multidrug-resistant, Extensively Drug-resistant, and Pandrug-resistant Pseudomonas aeruginosa Isolates from Burn Patients. Adv Biomed Res. 2017;6:74. doi: 10.4103/abr.abr_239_16 28706882 PMC5501067

[53] Kunz Coyne AJ, El Ghali A, Holger D, Rebold N, Rybak MJ. Therapeutic Strategies for Emerging Multidrug-Resistant Pseudomonas aeruginosa. Infect Dis Ther. 2022;11(2):661–82. doi: 10.1007/s40121-022-00591-2 35150435 PMC8960490

[54] Akinduti PA, George OW, Ohore HU, Ariyo OE, Popoola ST, Adeleye AI, et al. Evaluation of Efflux-Mediated Resistance and Biofilm formation in Virulent Pseudomonas aeruginosa Associated with Healthcare Infections. Antibiotics (Basel). 2023;12(3):626. doi: 10.3390/antibiotics12030626 36978493 PMC10044907

[55] Elmassry MM, Colmer-Hamood JA, Kopel J, San Francisco MJ, Hamood AN. Anti-Pseudomonas aeruginosa Vaccines and Therapies: An Assessment of Clinical Trials. Microorganisms. 2023;11(4):916. doi: 10.3390/microorganisms11040916 37110338 PMC10144840

[56] Adwani S, Desai UD, Joshi JMJ. Prevalence of pre-extensively drug-resistant tuberculosis (Pre XDR-TB) and extensively drug-resistant tuberculosis (XDR-TB) among pulmonary multidrug resistant tuberculosis (MDR-TB) at a tertiary care center in Mumbai. J J K I o M S U. 2016;5(3). 10.4103/lungindia.lungindia_182_22 PMC9894272 36695254

[57] Alarcon-Braga EA, Salazar-Valdivia FE, Estrada-Grossmann JM, Mendez-Guerra C, Pacheco-Barrios N, Al-kassab-Córdova AJA. Pre-extensively drug-resistant and extensively drug-resistant tuberculosis in Latin America and the Caribbean: A systematic review and meta-analysis. A J Infect Dis. 2024;52(3):349–57. 10.1016/j.ajic.2023.12.001 38061402

[58] Tasnim T, Tarafder S, Alam FM, Sattar H, Kamal SM. Pre-extensively drug resistant tuberculosis (Pre-XDR-TB) among pulmonary multidrug resistant tuberculosis (MDR-TB) patients in Bangladesh. J Tuberc Res. 2018;6(3):199–206.

[59] Daniel O, Osman E, Oladimeji O, Dairo OGJGAR. Pre-extensive drug resistant tuberculosis (Pre-XDR-TB) among MDR-TB patients in Nigeria. GAR JoM. 2013;2(2).

[60] Disratthakit A, Prammananan T, Tribuddharat C, Thaipisuttikul I, Doi N, Leechawengwongs M, et al. Role of gyrB Mutations in Pre-extensively and Extensively Drug-Resistant Tuberculosis in Thai Clinical Isolates. Antimicrob Agents Chemother. 2016;60(9):5189–97. doi: 10.1128/AAC.00539-16 27297489 PMC4997844

[61] Woldu MAJDM. Antimicrobial resistance in Ethiopia: current landscape, challenges, and strategic interventions. 2024;1(1):68.

[62] Hailegabriel EAJOHN. Managing antimicrobial resistance in Ethiopia: some lessons for other African countries. 2023;15(1).

[63] Tirfe M, Alemu A, Alemu W, Woldearegay M, Asfaw G, Gerba H, et al. A three years antimicrobials consumption in Ethiopia from 2017 to 2019: A cross- sectional study. PLoS One. 2023;18(4):e0284038. doi: 10.1371/journal.pone.0284038 37023072 PMC10079031

[64] Elema TB, Negeri AA, Verstraete L, Desta AF, Al-Mulla T, Goyol K, et al. Water, sanitation, and hygiene in selected health facilities in Ethiopia: risks for healthcare acquired antimicrobial-resistant infections. Front Public Health. 2024;12:1478906. 39687725 10.3389/fpubh.2024.1478906 PMC11647025

[65] Abosse JS, Megersa B, Zewge F, Eregno FE. Healthcare waste management and antimicrobial resistance: a critical review. J Water Health. 2024;22(11):2076–93. doi: 10.2166/wh.2024.232 39611670

[66] Gemeda BA, Amenu K, Magnusson U, Dohoo I, Hallenberg GS, Alemayehu G, et al. Antimicrobial Use in Extensive Smallholder Livestock Farming Systems in Ethiopia: Knowledge, Attitudes, and Practices of Livestock Keepers. Front Vet Sci. 2020;7:55. doi: 10.3389/fvets.2020.00055 32175334 PMC7055293

[67] WHO. Ethiopia intensifies battle against antimicrobial resistance amid global crisis. 2024. https://www.afro.who.int/countries/ethiopia/news/ethiopia-intensifies-battle-against-antimicrobial-resistance-amid-global-crisis

[68] Hailemichael T, Girma L, Fissiha P, Geteneh A, Kassa T. Correction: Isolation of virulent phages against multidrug-resistant acinetobacter baumannii recovered from inanimate objects of Jimma Medical Center, Southwest Ethiopia. BMC Infect Dis. 2023;23(1):894. doi: 10.1186/s12879-023-08915-4 38124032 PMC10734121

[69] Sada TS, Tessema TS. Isolation and characterization of lytic bacteriophages from various sources in Addis Ababa against antimicrobial-resistant diarrheagenic Escherichia coli strains and evaluation of their therapeutic potential. BMC Infect Dis. 2024;24(1):310. doi: 10.1186/s12879-024-09152-z 38486152 PMC10938718

[70] Amankwah S, Adisu M, Gorems K, Abdella K, Kassa T. Assessment of Phage-Mediated Inhibition and Removal of Multidrug-Resistant Pseudomonas aeruginosa Biofilm on Medical Implants. Infect Drug Resist. 2022;15:2797–811. doi: 10.2147/IDR.S367460 35668859 PMC9166914

[71] Gadisa E, Tadesse E. Antimicrobial activity of medicinal plants used for urinary tract infections in pastoralist community in Ethiopia. BMC Complement Med Ther. 2021;21(1):74. 33622320 10.1186/s12906-021-03249-7 PMC7903779

[72] NDMC NDMCH. Antimicrobial Resistance: The Subsequent Challenge of Ethiopia’s Health Care System. 2022. https://ndmc.ephi.gov.et/antimicrobial-resistance-the-subsequent-challenge-of-ethiopias-health-care-system/

[73] Assefa M, Amare A, Tigabie M, Girmay G, Setegn A, Wondmagegn YM, et al. Burden of multidrug-resistant bacteria among HIV-positive individuals in Ethiopia: A systematic review and meta-analysis. PLoS One. 2024;19(8):e0309418. doi: 10.1371/journal.pone.0309418 39186717 PMC11346931

[74] Geleta D, Abebe G, Tilahun T, Ahmed H, Workneh N, Beyene G. Prevalence and pathogen profiles of bacteremia in neonates hospitalized for clinical Sepsis in Ethiopia: a systematic review and meta-analysis. BMC Infect Dis. 2024;24(1):1424. doi: 10.1186/s12879-024-10312-4 39695487 PMC11654219

[75] Legese MH, Asrat D, Mihret A, Hasan B, Mekasha A, Aseffa A, et al. Genomic Epidemiology of Carbapenemase-Producing and Colistin-Resistant Enterobacteriaceae among Sepsis Patients in Ethiopia: a Whole-Genome Analysis. Antimicrob Agents Chemother. 2022;66(8):e0053422. doi: 10.1128/aac.00534-22 35876577 PMC9380574

[76] Sisay A, Kumie G, Gashaw Y, Nigatie M, Gebray HM, Reta MAJBID. Prevalence of genes encoding carbapenem-resistance in Klebsiella pneumoniae recovered from clinical samples in Africa: systematic review and meta-analysis. 2025;25(1):1–22. 10.1186/s12879-025-10959-7 PMC12007206 40251495

