# Supplementary material for: Emergence of extensively and pan-drug resistance in clinical bacterial isolates: A systematic scoping review from Ethiopian public health perspective
Source: PLoS Negl Trop Dis. 2025 Aug 28;19(8):e0013363. doi: 10.1371/journal.pntd.0013363 (PMC12393774; doi:10.1371/journal.pntd.0013363)
Supplement: S3 File — (DOCX) [file pntd.0013363.s003.docx]

**S3 File. Abbreviations**

AMR; Antimicrobial Resistance, MDR; Multidrug-resistant, XDR; Extensively drug-resistant, PDR; Pandrug-resistant, TB; Tuberculosis, Pre-XDR-TB; Pre-extensively drug-resistant TB, GARDP; Global Antibiotic Research and Development Partnership, NAP-AMR; National Action Plan for Antimicrobial Resistance, PRISMA-ScR; Preferred Reporting Items for Systematic Reviews and Meta-Analyses Extension for Scoping Reviews,PROSPERO; International Prospective Register of Systematic Reviews
